# Antibiotic use in pig farming and its associated factors in L County in Yunnan, China

**DOI:** 10.1002/vms3.390

**Published:** 2020-11-07

**Authors:** Jing Fang, Guodong Gong, Jingsong Yuan, Xiao Sun

**Affiliations:** ^1^ Institute for Health Sciences Kunming Medical University Kunming City China; ^2^ Disciplinary Inspection & Supervision Office the first Affiliated Hospital of Zhejiang University Hangzhou China; ^3^ Science and Technology Department Jiangxi Provincial Institute of Traditional Chinese Medicine Nanchang China; ^4^ Clinical Medicine School of BinZhou Medical University Binzhou China

**Keywords:** antibiotic use, behaviours, pig farming

## Abstract

China has a long history of pig rearing, and it currently raises and consumes approximately half of the pigs in the world. Major improvements have been made in pig farming in China in the last four decades with the growing application of new livestock farming technologies. Among the new improvements, the use of antibiotics in pig farming is a common but not well‐documented practise. In order to understand the behaviour of the farmers regarding antibiotic use in pig farming, we conducted a household survey in four townships of L County in Yunnan Province, China, during August 2014 and April 2015. In this survey, 404 farmer households were interviewed using a questionnaire. Among the farmers interviewed, 89% reported easy access to antibiotics, 83.7% reported experience of self‐purchasing antibiotics, and 40.3% reported that they often used antibiotics in pig farming mainly for the prevention and treatment of pig diseases. These farmers identified 20 antibiotics that they had used in pig farming 6 months before the survey. Of these, 11 and 8 antibiotics have been categorised under ‘critically important’ and ‘highly important’ antimicrobial groups, respectively, by the World Health Organization (WHO), and 12 and 8 have been categorised under the ‘Watch’ and ‘Access’ groups, respectively, as per the 2019 WHO AWaRe classification of antibiotics. Factors associated with the behaviour of self‐purchasing antibiotics included types of farms, sources of antibiotics, and previous experiences of pig diseases: those who were smallholders, buying antibiotics from veterinary drugstores and village vets, and whose pigs had suffered diseases previously were more likely to self‐purchase antibiotics for their pigs. Farmers who cleaned their pigsties less frequently and those whose pigs had suffered from diseases used antibiotics more frequently as compared to their peer farmers.

## Abstract 2

This survey conducted in a county in Yunnan Province of China finds out that 83.7% of the farmers reported ‘self‐purchasing’ antibiotics for their pigs and 40.3% of the farmers expressed that they ‘often’ use antibiotics in pig farming with the major purposes being prevention and treatment of pig diseases.

**Impacts**

- This survey, conducted in a county in the Yunnan Province of China, showed that 83.7% of the farmers reported self‐purchasing antibiotics for their pigs and 40.3% expressed that they often use antibiotics in pig farming mainly for the prevention and treatment of pig diseases.
- These farmers reported 20 antibiotics that they had used in pig farming in the last 6 months before the survey. Of these antibiotics, 11 and 8 have been categorised under ‘critically important’ and ‘highly important’ antimicrobial groups, respectively, by WHO, and 12 and 8 have been categorised under the ‘Watch’ and ‘Access’ groups, respectively, as per the 2019 WHO AWaRe classification of antibiotics.
- Factors associated with the behaviour of self‐purchasing antibiotics included types of farms, sources of antibiotics, and the experiences of previous pig diseases, and the factors associated with the farmers often using antibiotics for their pigs included the frequency of cleaning pigsty and previous pig diseases.

## 1 INTRODUCTION

China is the world's biggest meat producer by far, and it currently raises and consumes approximately half of the pigs in the world (Elliott, 2015). Moreover China has a long history of pig rearing (Cucchi et al., 2016; Kuo, 2013). In the last four decades, major improvements have been made in pig farming in rural China with the growing application of new livestock farming technologies, such as new breeds, feed, vaccination and veterinary drugs. The new technologies have greatly enhanced the productivity of pig farming by increasing the supply of pork to meet the increasing market demand. However, these practises have had profound implications, both positive and negative, for public health. The positive aspects include improved nutritional status of the population and increased income of pig farmers, whereas negative aspects include, but are not limited to, environmental pollution caused by unutilised pig manure and potential threats of zoonoses. The application of antibiotics in pig farming is an increasing but not well documented and regulated practise. Studies have revealed that samples of pig manure and soil collected from large swine farms in China contained diverse and abundant antibiotic resistance genes (Mu et al., 2015; Zhu et al., 2013), many of which were found in chicken and human faeces as well (Ma et al., 2015). While it is well acknowledged that antibiotics are widely used in pig farming in China (Wang et al., 2017), the behaviours of pig farmers regarding antibiotic use, particularly smallholder farmers, are neither well documented nor understood due to insufficient research. In order to regulate the use of antibiotics better in animal husbandry and to limit antimicrobial resistance, we undertook research in a county in the Yunnan Province of China to understand the behaviour of antibiotic use among pig farmers and its associated factors. This study was funded by the International Development Research Centre, Canada, and the Innovative Research Team of Yunnan Province (2019[6]).

## 2 MATERIALS AND METHODS

### 2.1 Study site

L County in the Yunnan Province of China was selected as the study site for several reasons. First, it is a poverty‐stricken county with a long history of pig rearing that has been promoted by the local government to alleviate poverty. Second, this county presents diverse forms of pig farming practises ranging from large‐scale modern pig farms to traditional smallholder pig rearing due to its mountainous geography and presence of ethnic minority groups. Third, the research team has better geographic access to this county because of its distance from Kunming (100 km), the capital city of Yunnan Province. In 2014, there were 13 townships and 160 administrative villages with a total population of 411.6 thousand, and 32% of them belonged to ethnic minority groups. Among the 13 townships in this county, four townships and eight administrative villages with two administrative villages per township were selected for this research. The selection was made after consulting with the County Animal Husbandry and Veterinary Bureau (CAHVB) to identify the townships and villages with the greatest number of pig farmers and highest density of pig population in the county. Thus, the selected townships and villages were not random, but purposefully chosen samples. All four townships and eight villages showed similar characteristics in terms of economic development level, culture, and veterinary services, and they were all located in the central part of this county. This county cannot be claimed as the representative of all counties in China, but it definitely represents some counties in the Yunnan Province showing similar geographic, socioeconomic and cultural conditions.

### 2.2 Household survey

The household survey was the main research method employed in this study. Interviews with local farmers, vets and staff members from the CAHVB were conducted as a supplementary method, with the findings used to design the survey questionnaire. Pig rearing requires inputs such as pigsty, feed, cleaning and disease prevention, and these factors affect the incidence of pig diseases and the subsequent treatment involving the use of veterinary medicines, including antibiotics. Therefore, we designed questions to investigate the types of pigsties, methods and frequency of pigsty cleaning, frequency of disinfection, sources of pig procurement, sources of drinking water, vaccinations and sources of feed. The main contents of the questionnaire included general demographic information of the pig farmers, pig farming size and rearing practises, previously encountered pig diseases, and the knowledge, accessibility and use behaviour of the farmers regarding antibiotics.

The sample size of the household survey was calculated using the statistical formula provided below, and the result was 384 farmer households. Considering incomplete questionnaires or missing data, we decided to expand the sample size to 450 households with 110–113 per township and 55–56 per administrative village. Prior to the formal survey, we pre‐tested the questionnaire between 17 and 19 july 2014. We investigated 50 pig farmers from two other villages of this county and found that many farmers could not recollect the name of the antibiotics that they had administered to their pigs in the last 6 months. Therefore, we consulted with local veterinary service providers and farmers to identify antibiotics that were most commonly used in the county, and selected 18 antibiotics to list in the questionnaire as an index. When we undertook the formal household survey, our trained investigators read the names of these 18 antibiotics one by one to the respondents and asked them whether they had used any of those antibiotics in the last 6 months, or if they had used any antibiotics apart from the 18 listed ones. Thus, we collected relatively accurate information from each surveyed farmer on antibiotic use.

$$n=\left[\frac{Z_{\alpha }^{2}P\left(1‐P\right)}{\delta^{2}}\right]$$

*α*: Type I error, *Z_α_* value: 1.96.*δ*: permitted error, defined as 0.05.*P*: positive rate, defined as 0.5n: sample size.

We planned to survey all pig farmers in the selected villages of the four townships, but not all farmers were available when we visited their houses for the survey. Hence, the surveyed farmer households formed a convenience sample, but they accounted for more than 80% of all pig farmers of the selected villages because we revisited those farmer houses at another time to capture those who had been missed previously. When starting the survey, the investigators explained the purpose and process of the survey, promised confidentiality of the participants’ identity information, and obtained oral informed consent from the farmers. Approximately 40–50 min were spent to complete one household questionnaire, with some even taking over an hour. A plastic washbowl costing around 1.5 USD was given to the surveyed farmers as a small gift to thank him/her for their time when the interview was concluded.

The survey was conducted in two different periods: August 2014 and April 2015. The major cause behind this time gap was that we could not complete the planned 450 household surveys in August 2014 and had to continue the survey another time. April 2015 was the time when both the research team and the CAHVB staff were available. Finally, 450 pig farmer household questionnaires were completed. These questionnaires were checked on the site every day to identify errors and missing data in a timely manner and make corrections whenever possible.

### 2.3 Data cleaning, entry and analysis

The completed questionnaires were brought to Kunming Medical University. Data were dual entered into EpiData 3.1 and crosschecked to ensure accuracy. Of the 450 completed questionnaires, 404 were valid and were analysed further, and the overall effective rate was 89.8% (404/450). Statistical Package for the Social Sciences Version 17.0 software was employed for statistical analysis. The number of pigs kept by the 404 farmers ranged between 1 and 1,138 heads (mean, 36 heads; median, 11 heads). We divided the 404 pig farmers into two groups, large‐scale farmers and smallholder farmers, based on the 2015 Data Compilation of National Agricultural Product Cost‐benefit issued by the Ministry of Agriculture, China. Large‐scale farmers were those who kept more than 30 heads, and smallholder farmers were those who kept fewer than 30 heads. We then compared the behaviours of antibiotic use of the farmers and the associated factors between the two groups using the chi‐square test or non‐parametric test, and analysed the factors that affected the farmers’ antibiotic use behaviours in pigs using binary multivariate logistic regression. Two behaviours were selected as dependent variables for the multivariate logistic regression analysis: self‐purchasing antibiotics and frequency of antibiotic use. Independent variables used to analyse the variable of ‘self‐purchasing antibiotics’ included participation in livestock rearing training or not, types of farm (large‐scale or smallholder), types of pig house (hygienic pig house or traditional pigsty^1^), methods of pig house disinfection (chemical disinfectants, quicklime or others), number of previous pig diseases, number of vaccines used, number of antibiotics used, purpose of using antibiotics, and sources of buying antibiotics. Independent variables used in the analysis of ‘frequency of antibiotic use’ included participation in livestock rearing training, frequency of cleaning pig houses, methods of pig house disinfection (chemical disinfectants, quicklime or others), number of previous pig diseases, number of vaccines used and number of antibiotics used. The criterion for including the variables in the multivariate logistic regression was *p* < .1 in the univariate analysis.

## 3 RESULTS

### 3.1 Basic demographic information of the respondents

Of the 404 surveyed farmers, 215 were female (53%) and 189 were male (46.8%). More than 70% (77.2%) of the surveyed farmers were aged 40 years and above, and 18.1% were aged 60 years and above. Han ethnicity, the majority group in China, accounted for 88.6% of the surveyed group, and the remaining (11.4%) belonged to ethnic minority groups. Approximately two‐thirds (59.9%) of the farmers had an education level of primary school or below, 34.2% attended middle school, and merely 5.9% had an education level of high school and above. Male farmers had a better education level than their female counterparts (χ^2^ = 12.784, *p* <  01), and majority (93.3%) of the farmers were married (Table 1).

**TABLE 1:** Basic demographic information of the 404 surveyed farmers

	Male		Female		Total		*χ^2^*	*p*
	*n*	%	*n*	%	*n*	%		
Education								
Illiteracy	19	10.1	45	20.9	64	15.8	12.784	.005^*^
Primary school	80	42.3	98	45.6	178	44.1		
Middle school	77	40.7	61	28.4	138	34.2		
High school and above	13	6.9	11	5.1	24	5.9		
Subtotal	189	100	215	100	404	100		
Age								
18~	10	5.3	24	11.2	34	8.4	11.093	.026^*^
30~	20	10.6	38	17.7	58	14.4		
40~	66	34.9	64	29.8	130	32.2		
50~	52	27.5	57	26.5	109	27.0		
60 and above	41	21.7	32	14.8	73	18.1		
Subtotal	189	100	215	100	404	100		
Ethnicity								
Han	169	89.4	189	87.9	358	88.6	0.228	.633
Minorities	20	10.6	26	12.1	46	11.4		
Subtotal	189	100	215	100	404	100		
Marriage								
Married	174	92.1	203	94.4	377	93.3	0.895	.344
Unmarried	15	7.9	12	5.6	27	6.7		
Subtotal	189	100	215	100	404	100		

### 3.2 General situation of pig rearing of the surveyed households

Among the 404 surveyed farmers, 86 (21.3%) were considered large‐scale farmers and 318 (78.7%) were smallholder farmers.

Table 2 shows that 81.2% of the surveyed farmers used hygienic pig houses, while 57.2% of them raised pigs that were self‐bred at home. Around two‐third (68.6%) of the farmers used both homemade and commercial factory‐produced pig feed, and 87.1% of them provided the pigs with tap water for drinking. More than 80% of the farmers (84.2%) cleaned their pig houses once in less than 7 days, and 65% of them used disinfectants. To summarize, the pig rearing practise of the 404 surveyed farmers exhibited a mixed pattern of traditional rearing methods integrated with modern techniques. For example, the farmers used traditional homemade pig feed together with commercial factory‐produced pig feed, and traditional pigsties co‐existed with hygienic pig houses. However, there were statistically significant differences between the large‐scale farmers and smallholders in terms of the types of pig house, methods of pig house cleaning, frequency of pig house cleaning, disinfection of pig house and sources of feed. Large‐scale farmers employed modern pig rearing techniques, including the use of hygienic pig houses, more frequent pig house cleaning, and use of chemical disinfectants and commercially produced feed, more commonly as compared to the smallholder farmers.

**TABLE 2:** Basic information of pig rearing of the 404 surveyed farmers

	Smallholders		Large‐scale farmers		Total		*χ^2^*	*p*
	*n*	%	*n*	%	*n*	%		
Types of pig house								
Traditional pigsty	76	23.9	0	0	76	18.8	25.316	.000^*^
Hygienic pig house	242	76.1	86	100.0	328	81.2		
Subtotal	318	100	86	100	404	100		
Methods of pig house cleaning								
Just remove manure	156	49.1	12	13.9	168	41.6	36.348	.000^*^
Wash with water	32	10.1	20	23.3	52	12.9		
Both	130	40.9	54	62.8	184	45.5		
Subtotal	318	100	86	100	404	100		
Frequency of cleaning pig house								
≤7days	256	80.5	84	97.7	340	84.2	14.971	.000^*^
>7days	62	19.5	2	2.3	64	15.8		
Subtotal	318	100	86	100	404	100		
Methods of disinfection								
Disinfectants	184	57.9	81	94.2	265	65.6	40.990	.000^*^
Quicklime	55	17.3	5	5.8	60	14.9		
Others	79	24.8	0	0	79	19.5		
Subtotal	318	100	86	100	404	100		
Source of pigs								
Home self‐bred	173	54.4	58	67.4	231	57.2	5.530	.063
Bought from market	129	40.6	23	26.7	152	37.6		
Both	16	5.0	5	5.8	21	5.2		
Subtotal	318	100	86	100	404	100		
Sources of feed								
Home made	16	5.0	1	1.2	17	4.2	15.010	.001^*^
Bought from market	73	23.0	37	43.0	110	27.2		
Both	229	72.0	48	55.8	277	68.6		
Subtotal	318	100	86	100	404	100		
Sources of drinking water								
Tap water	282	88.7	70	81.4	352	87.1	3.202	.074
Well water	36	11.3	16	18.6	52	12.9		
Total	318	100	86	100	404	100		

### 3.3 Antibiotic use in pig rearing by the surveyed farmers

#### 3.3.1 Farmers’ accessibility to antibiotics

Among the surveyed farmers, 89% acknowledged that it was easy for them to buy antibiotics for pigs, and 93.3% of them reported that they could buy antibiotics over‐the‐counter without a prescription issued by a vet (Tables 3 and 4).

**TABLE 3:** Farmers’ accessibility to antibiotics

	Easy			Ordinary		Difficult		Total		*Z*	*p*
	*n*		%	*n*	%	*n*	%	*n*	%		
Smallholder farmers	280	88.1		22	6.9	16	5.0	318	100	−1.599	.110
Large‐scale farmers	81	94.2		2	2.3	3	3.5	86	100		
Total	361	89.4		24	5.9	19	4.7	404	100		

**TABLE 4:** Needing a prescription issued by a vet to buy antibiotics

	Yes		No		Total		*χ^2^*	*p*
	*n*	%	*n*	%	*n*	%		
Smallholder farmers	20	6.3	298	93.7	318	100	0.372	.542
Large‐scale farmers	7	8.1	79	91.9	86	100		
Total	27	6.7	377	93.3	404	100		

Regarding sources of antibiotics, 54.8% and 26.5% of the farmers (total, 81.3%) reported that they mainly purchased antibiotics from local veterinary drugstores and village vets, respectively (Table 5). This suggests that despite the lack of a prescription, the purchasing behaviours suggested certain professional oversight if the sellers and vets were qualified veterinary professionals. However, the qualitative interview data revealed that most sellers at the local township and village veterinary drugstores were not qualified veterinary professionals, and the village vets were part‐time personnel with limited veterinary medicine training. Furthermore, 16.9% and 1.9% of the farmers reported that they bought antibiotics from human pharmacy shops and village clinics, respectively, to treat their sick pigs. This indicated the possibility of using antibiotics kept for humans to treat the pigs. Our household survey could not identify all antibiotics purchased by the farmers for the sick pigs from the human pharmacy shops and clinics. The few names frequently mentioned by farmers included, but were not limited to, penicillin, amoxicillin and oxytetracycline. Although these antibiotics are used in animal husbandry too, the Veterinary Drug Management Regulation issued by the Ministry of Agriculture, China in 2004 has clearly prohibited the use of human medicine, including human antibiotics, in animals. The qualitative interview data revealed that the main motivations for using human medicine for pigs included the perceived better quality of human medicine and dual use of human medicines for both human beings and animals after purchase. In some cases, farmers used the leftover antibiotics from a human disease treatment course of family members to treat the sick pigs.

**TABLE 5:** Sources of antibiotics used by farmers in pig rearing

	Veterinary drugstore		Village vets		Pharmacy		Village clinic		Total		*χ^2^*	*p*
	*n*	%	*n*	%	*n*	%	*n*	%	*n*	%		
Smallholders	220	51.6	122	28.6	75	17.6	9	2.1	426	100	**8.934**	.03^*^
Large‐scale farmers	72	67.3	19	17.8	15	14.0	1	0.9	107	100		
Total	292	54.8	141	26.5	90	16.9	10	1.9	533	100		

##### Farmers’ behaviours regarding self‐purchasing antibiotics

In this study, self‐purchasing antibiotics was defined as farmers purchasing antibiotics for pigs over‐the‐counter, without consulting a vet or presenting a prescription issued by qualified vets. Of the 404 surveyed farmers, 83.7% reported self‐purchasing antibiotics, suggesting that this behaviour was common among the surveyed farmers.

There were no statistically significant differences in the behaviour of self‐purchasing antibiotics between the farmer groups with respect to sex, age, education level, ethnicity, training on livestock rearing and years of pig raising (data not presented). Table 6 shows that more large‐scale farmers (93%) reported this experience as compared to smallholder farmers (81.1%). Furthermore, farmers who used hygienic pig houses and chemical disinfectants for pig house disinfection reported this behaviour more than those who used traditional pigsties and quicklime for disinfection. However, these differences could have been caused by confounders.

**TABLE 6:** Farmers’ behaviours of self‐purchasing antibiotics by farm types, pig houses, methods and frequency of pig house cleaning and disinfection

	Yes		No		Total		*χ^2^*	*p*
	*n*	%	*n*	%	*n*	%		
Types of pig farms								
Smallholders	258	81.1	60	18.9	318	100	7.003	.008^*^
Large‐scale farmers	80	93.0	6	7.0	86	100		
Subtotal	338	83.7	66	16.3	404	100		
Types of pig house								
Traditional pigsty	57	75.0	19	25.0	76	100	5.140	.023^*^
Hygienic pig house	281	85.7	47	14.3	328	100		
Subtotal	338	83.7	66	16.3	404	100		
Methods of cleaning								
Just remove manure	138	82.1	30	17.9	168	100	0.692	.707
Wash with water	43	82.7	9	17.3	52	100		
Both	157	85.3	27	14.7	184	100		
Subtotal	338	83.7	66	16.3	404	100		
Frequency of cleaning								
≤7days	290	85.0	51	15.0	341	100	3.050	.081
>7days	48	76.2	15	23.8	63	100		
Subtotal	338	83.7	66	16.3	404	100		
Disinfection								
Disinfectants	231	87.2	34	12.8	265	100	8.626	.013^*^
Quicklime	49	81.7	11	18.3	60	100		
Others	58	73.4	21	26.6	79	100		
Subtotal	338	83.7	66	16.3	404	100		

We performed binary multivariate logistic regression to identify factors that affected the farmers’ antibiotic self‐purchasing behaviours. The results showed that farm types, sources of antibiotics and previous pig diseases were linked with self‐purchasing behaviour. Smallholder farmers, farmers who bought antibiotics from veterinary drugstore/village vets, and those whose pigs had previous diseases were more likely to self‐purchase antibiotics for pigs as compared to the others (Tables 7 and 8).

**TABLE 7:** Dependent and independent variables of multivariate logistic regression

Dependent variables	Independent variables*	Values of variables
Self‐purchasing antibiotics	Participation in livestock rearing training	No = 0, Yes = 1
No = 0	Types of farm	Smallholders = 0, large‐scale farmers = 1
Yes = 1	Types of pig house	Traditional pigsty = 0, hygienic pig house = 1
	Methods of disinfection	quicklime = 1, disinfectants = 2, others = 3
	Number of previous pig diseases	Reported number
	Number of vaccines used	Reported number
	Number of antibiotics used	Reported number
	Purpose of antibiotic use	Prevention = 0, treatment = 1
	Sources of buying antibiotics	Pharmacy/village clinics = 0, veterinary drugstores/village vets = 1
Frequency of antibiotic use	Participation in livestock rearing training	No = 0, Yes = 1
Not often use = 0 Often use = 1	Frequency of cleaning pig house	≤7 days = 0,>7 days = 1
	Methods of disinfection	quicklime = 1, disinfectants = 2, others = 3
	Number of previous pig diseases	Reported number
	Number of vaccines used	Reported number
	Number of antibiotics used	Reported number

* : Criteria for including those independent variables into the multivariate logistic regression analysis was their *p* value < .1 in the univariate analysis.

**TABLE 8:** Odds ratios for risk factors linked to the self‐purchasing antibiotics for pigs

Dependent variable	Independent variables	Β	χ^2^	OR(95%CI)	*p*
Self‐purchasing antibiotics	**Farm types**				
	Large‐scale farmers	−1.205	7.076	0.300 (0.123, 0.728)	.008^*^
	Smallholders (reference)	0	—	—	—
	**Sources of antibiotics**				
	Veterinary drugstores/village vets	1.857	12.114	6.404 (2.251,18.221)	.001^*^
	Pharmacy/village clinics(reference)	0	—	—	—
	**Previous pig diseases**	0.219	5.271	1,244 (1.033,1.500)	.022^*^

#### 3.3.2 Farmers’ behaviours of using antibiotics in pig rearing

Table 9 shows 40.3% of the surveyed farmers expressed that they often used antibiotics in pig farming. Farmers who received training on livestock raising provided by local animal husbandry and veterinary systems, cleaned their pig house more frequently, used chemical disinfectants for pig house disinfection, and had encountered pig diseases were more likely to report the frequent use of antibiotics than farmers who did not. However, these differences might be caused by confounders. There were no differences in the frequency of antibiotic use among the farmers of different sex, age, education level, farm types and years of raising pigs (Table 9).

**TABLE 9:** Reported frequency of using antibiotics in pig rearing

	Often		Sometimes		Never		Total		*Z*	*p*
	*n*	%	*n*	%	*n*	%	*n*	%		
Sex										
Male	82	43.4	90	47.6	17	9.0	189	100	−0.473	.636
Female	81	37.7	114	53.0	20	9.3	215	100		
Subtotal	163	40.3	204	50.5	37	7.9	404	100		
Education										
Illiteracy	25	39.1	32	50.0	7	10.9	64	100	0.884	.829
Primary school	74	41.6	88	49.4	16	9.0	178	100		
Middle school	56	40.6	71	51.4	11	8.0	138	100		
High school and above	8	33.3	13	54.2	3	12.5	24	100		
Subtotal	163	40.3	204	50.5	37	9.2	404	100		
Age										
18~	10	29.4	19	55.9	5	14.7	34	100	7.585	.108
30~	18	31.0	38	65.5	2	3.5	58	100		
40~	50	38.5	62	47.7	18	13.9	130	100		
50~	49	45.0	53	48.6	7	6.4	109	100		
60 and above	36	49.3	32	43.8	5	6.9	73	100		
Subtotal	163	40.3	204	50.5	37	9.2	404	100		
Training on livestock										
Received	48	46.6	53	51.5	2	1.9	103	100	−2.264	.024^*^
Not received	115	38.2	151	50.2	35	11.6	301	100		
Subtotal	163	40.3	204	50.5	37	9.2	404	100		
Years of raising pigs										
<20	72	36.9	108	55.4	15	7.7	195	100	−0.837	.403
≥20	91	43.5	96	45.9	22	10.5	209	100		
Subtotal	163	40.3	204	50.5	37	9.2	404	100		
Types of farms										
Smallholders	124	39.0	161	50.6	33	10.4	318	100	−1.458	.145
Large‐scale farmers	39	45.3	43	50.0	4	4.7	86	100		
Subtotal	163	40.3	204	50.5	37	9.2	404	100		
Frequency of cleaning										
≤7days	146	42.8	163	47.8	32	9.4	341	100	−1.901	.050^*^
>7days	17	27.0	41	65.1	5	7.9	63	100		
Subtotal	163	40.3	204	50.5	37	9.2	404	100		
Methods of disinfection										
Disinfectants	117	44.2	124	46.8	24	9.1	265	100	9.142	.010^*^
Quicklime	18	22.8	54	68.4	7	8.9	79	100		
Others	28	46.7	26	43.3	6	10.0	60	100		
Subtotal	163	40.3	204	50.5	37	9.2	404	100		
had pig diseases										
Yes	127	46.5	132	48.4	14	5.1	273	100	−4.483	.000^*^
No	36	27.5	72	55.0	23	17.6	131	100		
Subtotal	163	40.3	204	50.5	37	9.2	404	100		

Using the frequency of antibiotic use as a dependent variable, we performed binary multivariate logistic regression to identify factors that affected this behaviour. The results showed two independent variables were included in the model at *p* < .05: frequency of cleaning pig house and previous pig diseases. Farmers who cleaned their pig houses less frequently were more likely to use antibiotics than those who cleaned them more frequently. Similarly, farmers whose pigs had suffered from diseases were more likely to use antibiotics than those whose pigs had not (Tables 7 and 10).

**TABLE 10:** Odds ratios for risk factors linked to the frequency of antibiotic use in pig farming

Dependent variable	Independent variables	Β	χ^2^	OR( 95%CI)	*p*
Frequency of antibiotics use	Frequency of cleaning pig house				
	>7days	0.737	5.771	2.090 (1.145, 3.813)	.016^*^
	≤7天 (reference)	0	—	—	—
	Number of previous pig diseases	0.143	5.981	1.154 (1.029,1.294)	.014^*^

In this study, 62% of the farmers stated that the purpose of using antibiotics was to treat pig diseases, 35.7% claimed that their use of antibiotics was to prevent pig diseases, and only 2.3% of the farmers said they used the drug to promote pig growth (Table 11). Thus, treatment and prevention of pig diseases were the two major purposes of antibiotic use reported by the surveyed farmers. There was no statistically significant difference between the large‐scale and smallholder farmers regarding the purpose of using antibiotics. However, there was a statistically significant difference between the male and female farmers (χ^2^ = 3.962, *p* < .05). More male farmers used antibiotics for disease prevention, whereas more female farmers used antibiotics for disease treatment. There were no differences in the purpose of using antibiotics among the two farmer groups with respect to age, education levels and years of raising pigs (Table 11).

**TABLE 11:** Purpose of using antibiotics in pig raising*

	Prevent diseases		Treat diseases		Promote growth		Total		*χ^2^*	*p*
	*n*	%	*n*	%	*n*	%	*n*	%		
Sex										
Male	72	39.3	104	56.8	7	3.8	183	100	3.962	.047*
Female	66	32.4	136	66.7	2	1.0	204	100		
Subtotal	138	35.7	240	62.0	9	2.3	387	100		
Education										
Illiteracy	16	29.1	39	70.9	0	0	55	100	3.457	.326
Primary school	59	35.8	103	62.4	3	1.8	165	100		
Middle school	53	36.6	87	60.0	5	3.4	145	100		
High school	10	45.5	11	50.0	1	4.5	22	100		
Subtotal	138	35.7	240	62.0	9	2.3	387	100		
Age										
18~	14	42.4	18	54.5	1	3.0	33	100	6.281	.179
30~	27	44.3	33	54.1	1	1.6	61	100		
40~	34	28.3	83	69.2	3	2.5	120	100		
50~	35	32.7	69	64.5	3	2.8	107	100		
60 and above	28	42.4	37	56.1	1	1.5	66	100		
Subtotal	138	35.7	240	62.0	9	2.3	387	100		
Type of farms										
Smallholders	99	34.6	182	63.6	5	1.8	286	100	1.222	.269
Large‐scale farmers	39	38.6	58	57.4	4	4.0	101	100		
Subtotal	138	35.7	240	62.0	9	2.3	387	100		
Training										
Yes	48	40.3	68	57.1	3	2.5	119	100	1.732	.188
No	90	33.6	172	64.2	6	2.2	268	100		
Subtotal	138	35.7	240	62.0	9	2.3	387	100		
Years of pig raising										
<20	69	37.1	112	60.2	5	2.7	186	100	0.493	.483
≥20	69	34.3	128	63.7	4	2.0	201	100		
Subtotal	138	35.7	240	62.0	9	2.3	387	100		

* : We combined farmers who reported disease prevention with farmers who reported growth promotion to allow sufficient counts in each grid and performed chi‐square test.

Moreover we used three hypothesised conditions to explore the farmers’ use of antibiotics further, namely when pigs had cold, fever and diarrhoea, and whether the farmers would use antibiotics to treat these conditions. The results revealed that the majority of smallholder and large‐scale farmers (78.3%–84.9%) reported that they would use antibiotics for the aforementioned conditions, and there was no statistically significant difference between the two groups (Table 12). Although it is difficult to evaluate the appropriateness of using antibiotics for those pig symptoms by asking a simple question, cold, fever and diarrhoea in pigs may not be caused by bacterial infection, and thus, using antibiotics would be ineffective. This implies a tendency for the misuse or overuse of antibiotics in pigs among the surveyed farmers.

**TABLE 12:** Antibiotic use for the hypothesised three pig ill conditions

	Smallholders		Large‐scale farmers		Total		*χ^2^*	*p*
	*n*	%	*n*	%	*n*	%		
Pigs have cold								
Use	249	78.3	70	81.4	319	79.0	0.390	.532
Not use	69	21.7	16	18.6	85	21.0		
Subtotal	318	100	86	100	404	100		
Pigs have fever								
Use	250	78.6	71	82.6	321	79.5	0.644	.422
Not use	68	21.4	15	17.4	83	20.5		
Subtotal	318	100	86	100	404	100		
Pigs diarrhoea								
Use	258	81.1	73	84.9	331	81.9	0.644	.422
Not use	60	18.9	13	15.1	73	18.1		
Subtotal	318	100	86	100	404	100		

#### 3.3.3 Major antibiotics used by farmers in pig rearing

Using the household survey, we investigated the antibiotics used by the farmers in pig rearing in the 6 months prior to the survey. The use of 20 antibiotics was reported, with oxytetracycline, penicillin, amoxicillin, cefoperazone, norfloxacin, ceftriaxone, ofloxacin, cefradine, chloramphenicol and sulfadiazine ranking in the top 10 as reported by 213 (52.7%), 182 (45.1%), 156 (38.6%), 82 (20.3%), 78 (19.3%), 75 (18.6%), 73 (18.1%), 64 (15.8%), 40 (9.9%) and 39 (9.6%) of the 404 surveyed farmers, respectively (Table 13). These antibiotics were the most commonly used ones by the surveyed farmers in pig rearing. The other 10 antibiotics used are also presented in Table 13. We sorted the 20 antibiotics into nine different classes based on their chemical structures. Table 14 presents the nine classes of antibiotics with the class of penicillin ranking the first (mentioned by 338 farmers), followed by tetracyclines (mentioned by 223 farmers).

**TABLE 13:** Antibiotics reported use by farmers in pig rearing

Antibiotics	No. of farmers who reported using	%	WHO AWaRe classification
Oxytetracycline	213	52.7%	Watch
Penicillin	182	45.1%	Access
Amoxicillin	156	38.6%	Access
Cefoperazone	82	20.3%	Watch
Norfloxacin	78	19.3%	Watch
Ceftriaxone	75	18.6%	Watch
Ofloxacin	73	18.1%	Watch
Cefradine	64	15.8%	Access
Chloramphenicol	40	9.9%	Access
Sulfadiazine	39	9.7%	Access
Lincomycin	38	9.4%	Watch
Ciprofloxacin	30	7.4%	Watch
Kanamycin	29	7.2%	Watch
Erythromycin	26	6.4%	Watch
Streptomycin	19	4.7%	Watch
Gentamicin	17	4.2%	Access
Levomycin	16	3.9%	Watch
Sulfaoxazole	15	3.7%	Access
Chlortetracycline	10	2.5%	Watch
Tetracycline	1	0.3%	Access

**TABLE 14:** Antimicrobial classes used by farmers in pig rearing

Classes of antibiotics	No. of farmers reporting use	%
Penicillins	338	83.7
Tetracyclines	223	55.2
Cephalosporins	221	54.7
Quinolones	188	46.5
Sulfonamides	54	13.4
Aminoglycosides	48	11.9
Amide alcohols	41	10.2
Link amine	38	9.4
Macrolides	26	6.4

#### 3.3.4 Antibiotic knowledge of the surveyed farmers

We designed 15 questions in the household survey questionnaire to test the farmers’ antibiotic knowledge. Table 15 shows that farmers had limited antibiotic knowledge. Among the 15 questions, the lowest and highest correct answer rates were between 5.4%–74%, wherein only 23.2% of farmers knew the concept of antibiotics, 30.2% farmers were aware of antimicrobial resistance and 5.4% knew the regulation that buying antibiotics needs a prescription issued by a vet. We divided the 404 farmers into two groups based on their antibiotic knowledge level: higher knowledge group (correct answers for 9 or more of the 15 questions, 60%), and low knowledge group (correct answers for 8 or less of the 15 questions). Next, we compared the behaviours regarding ‘self‐purchasing antibiotics’ and ‘frequency of antibiotic use’ of the two groups using the chi‐square test. There were no statistically significant differences found between the two groups (*p* > .1 for both; data not shown). Thus, the surveyed farmers’ antibiotic knowledge was not included as an independent variable in the multivariate logistic regression analysis.

**TABLE 15:** Antibiotic knowledge of the 404 surveyed farmers

Questions	Correct answer		Incorrect answer	
	*n*	%	*n*	%
Concept of antibiotics	76	23.2	328	76.8
Amoxicillin is an antibiotics	139	34.4	265	65.6
Antibiotics kill virus	59	14.6	345	85.4
Antibiotics kill bacteria	107	26.5	297	73.5
Antibiotics enhance animals’ immune capacity	186	46.0	218	54.0
Expensive antibiotics have better effects	218	54.0	186	46.0
Greater quantity of antibiotics produce better effects	299	74.0	105	26.0
Stopping time of an antibiotic treatment course	30	7.5	374	92.5
Withdrawal period	151	37.4	253	62.6
Antimicrobial resistance	122	30.2	282	69.8
Residues of antibiotics	116	28.7	288	71.3
Harmful effects of antibiotic misuse and overuse	194	48.0	210	52.0
Antibiotic residues in meat products can enter human body	251	62.1	153	37.9
Meat products containing antibiotic residues are harmful to human beings	294	72.8	110	27.2
Purchasing antibiotics needs prescription from vets	22	5.4	382	94.6

## 4 Discussion

### 4.1 Unrestricted antibiotics use in pig farming and weak regulation

Our survey data showed that both large‐scale and smallholder farmers in this county had easy access to antibiotics, and 40% of them often used antibiotics in pig farming, which is similar to that in other developing countries (Dang et al., 2013). The data revealed the weak enforcement of the regulatory policy issued by the Ministry of Agriculture, China in 2013 and effected in 2014, ‘The Management Regulation of Veterinary Prescription Drugs and Non‐prescription Drug', which stipulates that customers need to present a prescription issued by a vet when purchasing antibiotics for animals. More than 90% of the surveyed farmers reported that they did not have to present a prescription when purchasing antibiotics. Interviews with veterinary drug sellers showed that they also did not comply with the regulations; instead, certain actively recommended drugs, including antibiotics, were sold to farmers who were looking for treatment for their pigs without asking for a prescription. Some pig‐feed sellers sold antibiotics to farmers in order to prevent diarrhoea and other diseases that may occur after eating the feed, particularly diarrhoea that may occur at certain production stages of pigs, for example, moving from weaners to growers. While pig farming has been controlled or even banned in some areas of China in order to control environmental pollution caused by animal farming (Li, 2013; State Council, 2016), pig farming is encouraged in this county by the government as a way of income generation and poverty alleviation, and the pig population density has increased as compared to the past. Larger pig populations have been raised in a narrow space with limited hygienic conditions, and the risks of pig diseases are increasing. Hence, antibiotics play an important role in the prevention and treatment of pig diseases. If farmers do not have good access to antibiotics, the pigs may be at a higher risk of dying from diseases, which would lead to economic losses. This could explain the weak enforcement of the policy and the dilemma faced by the government. While it is imperative to enforce the regulation, measures need to be taken to ensure the farmers’ access to antibiotics and their use under the guidance of qualified veterinary professionals. Further research is needed to monitor the impact of this regulation on pig morbidity and mortality and farmers’ income when fully implemented.

### 4.2 Purpose of antibiotic use

The main purpose of using antibiotics in pig farming as reported by farmers in our survey sample was to prevent and treat pig diseases to avoid the economic loss caused by pig deaths, which is different from the behaviour of using antibiotics as a growth promoter. The results of multivariate logistic regression also suggest that farmers whose pigs had diseases in the past were more likely to self‐purchase and use antibiotics. In fact, most of the surveyed farmers were not aware of the growth‐promoting effect of antibiotics. Notably, when pigs show disease symptoms such as fever and not eating the feed, or there is a rumour of pig disease epidemic, majority of the farmers put antibiotics into the feed or drinking water to treat the symptoms or prevent the disease. Chen et al.’s study conducted in Jiangsu Province, China on the use of antimicrobials by pig farmers revealed that poorly educated, older, male farmers with over 10 years of pig production experience on small‐ and medium‐scale farms were most likely to engage in improper veterinary drug use (Chen et al., 2016), which is similar to our findings in this study. Since most farmers in our study were older than 40 years with little school education and training on livestock husbandry, along with easy accessibility to antibiotics, their use and misuse of antibiotics in pig farming were very likely. This finding suggests that if we want to reduce the farmers’ use and misuse of antibiotics in pig farming in this setting, we need to find alternative ways other than antibiotics to help them in the prevention and control of pig diseases and the associated economic loss.

### 4.3 Classification of antibiotics most commonly used by the surveyed farmers

Farmers identified 20 antibiotics that they had used in pigs in the 6 months before the survey. Although the magnitude of using these antibiotics, including frequencies and dosages, was unknown due to the limitations of this survey, it is worth noting that 11 and 8 of those antibiotics have been categorised as ‘critically important’ and ‘highly important’ antimicrobials, respectively, by the WHO in 2013. Moreover 10 were categorised as ‘prioritisation of critically important antibiotics’ (WHO, 2013). Among the nine classes of antibiotics, the quinolones and macrolides classes to which four of the 20 listed antibiotics belonged, were listed by the WHO using three criteria in 2013 as the ‘highest priority critically important antimicrobials’ for which risk management strategies are needed most urgently. Furthermore, 12 and 8 of the 20 antibiotics featured in the ‘Watch’ and ‘Access’ groups, respectively, as per the 2019 WHO AWaRe classification of antibiotics (WHO, 2019). Therefore, further research is warranted to confirm the antibiotics used and to monitor the antimicrobial resistance associated with their use.

### 4.4 Use of human antibiotic products to treat pigs

Both animals and humans need antibiotics to treat bacterial infections, but certain antibiotics usage is indicated for human beings only (WHO, 2013). The regulation of antibiotic use in human and veterinary medicine varies from country to country, with increasing countries banning or tightening the use of antibiotics as growth promoters. Although many antibiotic‐active pharmaceutical ingredients are used in both human and animal medicines, the Act on the Veterinary Drug Administration issued in China in 2004 has banned the use of human antibiotic products in animals. Our survey revealed that 18.8% of the surveyed farmers, both smallholder and large‐scale, reported the experience of buying antibiotics from human pharmacies or village clinics for pigs, indicating the use of human antibiotics in animals. Other studies conducted in China confirmed that the use of human antibiotic products to treat sick animals may be a common practise among farmers (Li, 2008; Ning et al., 2018; Ren, 2015; Wu et al., 2013). Although the farmers’ main purpose of using human antibiotic products to treat sick pigs was pursuing better efficacy, this behaviour not only violates the Act, but also increases the risk of antimicrobial resistance to human antibiotic products. This is because bacteria with antimicrobial genes developed in animals consumed as food could be transferred to humans via food chains and other channels (Landers et al., 2012). In fact, both human and animal health requires antibiotics, and many antibiotics are common between the human healthcare and veterinary sectors. Therefore, we need to develop strategies to find a good balance between the two sectors, and more importantly, contain the development of antimicrobial resistance.

### 4.5 Limited knowledge of antibiotics and antimicrobial resistance among farmers

The surveyed farmers exhibited poor knowledge of antibiotics, and they used antibiotics indiscriminately like the other veterinary medicines. More than two‐thirds of the farmers did not know the concept of antimicrobial resistance. Given that most farmers had only primary school education, it is not surprising that they had poor knowledge of antibiotics and low awareness regarding antimicrobial resistance. Notably, a survey conducted in Switzerland revealed that Swiss pig farmers were less aware of the risks of antibiotic usage in pig husbandry (Visschers et al., 2014). This suggests that farmers in both developed and developing countries need to be educated on the risks and consequences of using antibiotics in animal husbandry. Alternatively, it suggests that education does matter as it is overridden by economics.

### 4.6 Limitations

This research mainly relies on the farmers’ reporting of their antibiotic use behaviours in pig rearing in the 6 months before the survey; thus, its results are subject to recall bias. Additionally, as non‐professionals, farmers were not familiar with the names of different antibiotics and might not have been able to recollect the names of the antibiotics used accurately. Although we addressed this issue by reading out 18 antibiotic names to the farmers one by one as an index during the survey, under or misreporting of antibiotic use could have occurred. Furthermore, this research could not quantify the dosage or amount of antibiotics used in local pig farming. The magnitude of antibiotic use might be underrepresented because this research focussed on the farmers’ use behaviours and did not cover the antibiotics contained in the commercially produced feed or those used by local vets who visited the farms to treat sick pigs. Nevertheless, this research laid down a basis for future research. Further research is needed to explore ways that can more accurately capture the behaviours of farmers, antibiotics and their quantities used in such settings, and role of veterinary drugstores and vets at the grassroots level, and to monitor the antimicrobial resistance associated with such using behaviours.

## 5 CONCLUSION

It has been increasingly acknowledged that antibiotics used in animals reared for food contribute to the development of antimicrobial resistance. Pig farming is socially, economically, as well as politically important in China, given the significant role that pork plays in the diet of Chinese people. Millions of pigs raised annually in China by millions of farmers with various modern animal husbandry technical inputs, including antibiotics, will have profound impacts on antimicrobial resistance and other public health issues. This research sheds some light on the behaviours of pig rearing farmers regarding antibiotic use in a county in Yunnan. Although the term ‘large‐scale farmers’ was used in this research, most pig farms in this county were not actual large‐scale factory‐style pig production systems that exist in developed countries or more developed parts of China. However, antibiotics were used widely and frequently by large number of small‐scale pig farmers in this county. Their behaviours regarding antibiotic use were more diverse than that of large‐scale factory‐style pig production systems. The main purpose of using antibiotics by the farmers was the prevention and treatment of pig diseases, which is different from the purpose of growth promotion that has been practised in many large‐scale pig factories. However, the majority of antibiotics used by these pig farmers fall under the groups of ‘critically’ and ‘highly important’ antimicrobials listed by WHO, and 60% of the 20 listed antibiotics fall under the ‘Watch’ group of the 2019 WHO AWaRe classification of antibiotics. To what extent the antibiotic use in this pig farming system contributes to antimicrobial resistance and antibiotic residues in pork products and the environmental pollution is unclear. Further research is warranted to explore these questions.

## CONFLICT OF INTEREST

The authors declare no conflict of interest.

## AUTHOR CONTRIBUTION

Jing Fang: Conceptualization; Data curation; Formal analysis; Funding acquisition; Investigation; Methodology; Project administration; Resources; Supervision; Validation; Writing‐original draft; Writing‐review & editing. GuoDong Gong: Data curation; Formal analysis; Investigation; Methodology; Validation. JingSong Yuan: Data curation; Investigation. Xiao Sun: Data curation; Investigation.

### PEER REVIEW

The peer review history for this article is available at https://publons.com/publon/10.1002/vms3.390.

## ENDNOTE

^1^ Pig farming has a long history in this county. In the past, almost every farmer household raised several pigs, and they kept pigs in simple shelters that were usually a part of the farmhouse located close to the toilet in order to collect pig faeces together with human waste for fertilizer. Hence, the pig rearing condition was unhygienic, and this type of pigsty was called a traditional pigsty. In order to reduce pig morbidity/mortality and promote productivity, the agricultural extension workers have promoted ‘hygienic pigsty’, that is a pigsty with better hygienic conditions, such as a separate pig house with cement flooring and walls that is easy to clean.
